# *Marsupella lusitanica* (Gymnomitriaceae, Marchantiophyta), a New Species of Sect. *Ustulatae* from Mountain Ranges of Portugal

**DOI:** 10.3390/plants12071468

**Published:** 2023-03-27

**Authors:** Ronald D. Porley, David Bell, Jan Kučera

**Affiliations:** 1 Cerca dos Pomares, CxP 409M, 8670-052 Aljezur, Portugal; 2 Royal Botanic Garden Edinburgh, 20a Inverleith Row, Edinburgh EH3 5LR, UK; 3 Faculty of Science, University of South Bohemia, Branišovská 1760, 370 05 České Budějovice, Czech Republic

**Keywords:** *Marsupella*, Portugal, European liverwort endemic, ITS, *trnF–trnT*, integrative taxonomy

## Abstract

A new species of *Marsupella* sect. *Ustulatae* Müll. Frib. ex R.M. Schust. is described following an integrated morphological and molecular–phylogenetic study which examined the recently found dioicous plants growing epilithically on acidic substrates in several mountain ranges of Portugal between Peneda-Gerês in the north and Serra da Monchique in the extreme south. Employed molecular markers (plastid *trnF–trnT* region and nuclear ribosomal ITS) confirmed the distinctness of the lineage from other currently recognized species in the section, and furthermore, previously neglected diversity within *M. sprucei* (Limpr.) Bernet was signaled. Although not yet confirmed outside Portugal, the newly reported species is probably not rare in the region and has likely been overlooked as *M. funckii* (F. Weber & D. Mohr) Dumort. or *M. profunda* Lindb. in the past.

## 1. Introduction

The predominately Northern Hemisphere liverwort family Gymnomitriaceae currently includes nine genera [1], of which *Marsupella* Dumort. is the most speciose, with thirty-three species worldwide [1,2,3,4], seven of which are recently described and two representing infraspecific taxa elevated to species rank [2,3,4]. In Europe, there are currently sixteen accepted species [5], and in Portugal, six species are reported [6]. Many species have been synonymized over time, though it is likely some will be resurrected as further molecular data inform taxonomic recircumscriptions, as in the case of *Marsupella aquatica* (Lindenb.) Schiffn. [7] or, more recently, *M. patens* (N. Kitag.) Bakalin & Fedosov and *M. vermiformis* (R.M. Schust.) Bakalin & Fedosov [3].

During a decade of bryological exploration by R.D. Porley of Serra de Monchique, Algarve, Portugal, the most south-westerly located mountain range in continental Europe, numerous *Marsupella* collections were made. Most of these were determined as *M. emarginata* (Ehrh.) Dumort., but a few collections, mostly from the summit of Fóia, the highest peak at 902 m a.s.l., but also from the north flank, clearly belonged to a different plant characterized by closely appressed flat glossy brownish-black patches and leaves with plane leaf margins and mostly subacute to obtuse leaf lobes. These plants initially called to mind *M. profunda* Lindb., a European endemic known from further north in mainland Portugal, England and Macaronesia [8]. Subsequent microscopic study, however, indicated a subtle difference in the angle between the leaf lobes, mostly 60–90° in the Monchique plants, opposed to 40–60° in *M. profunda* [9], but more remarkable was that the Monchique plants appeared to be dioicous, whereas *M. profunda* is paroicous. Material was then sent to D.G. Long (RBG Edinburgh, Edinburgh, UK) and N.G. Hodgetts (Cuillin Views, Isle of Skye, UK), who both agreed that the Portuguese plants did not accord with *M. profunda* and that the plants were ostensibly dioicous. To resolve the identity of the Portuguese plants, R.D. Porley sent material to J. Kučera, who sequenced them to reveal the molecular affinities using the sequence data publicly accessible in GenBank and unpublished data from a DNA barcoding study on rare British bryophytes [10]. The comparison of sequence data for nuclear ribosomal ITS region and chloroplast *trnL–trnF* and *trnH–psbA* showed that the plants did not match *M. profunda* or any other previously sequenced species, except for two at that time unreleased molecular accessions (included in analyses here), retrieved from specimens collected by D. Bell and D.G. Long during a visit to northern Portugal (*Bell* 251 and *Bell* 247, respectively), and tentatively named *Marsupella sprucei* (Limpr.) Bernet [10]. Later examination of the specimens confirmed that the plants were apparently dioicous (*M. sprucei* is paroicous), with separate male and female plants observed in both collections. Three additional specimens of the unknown plant, confirmed later by molecular data, were found among Portuguese collections of *Marsupella funckii* (F. Weber & D. Mohr) Dumort. by J. Kučera, held in herbarium CBFS. Indeed, the prominent Czech hepaticologist Jiří Váňa (1940–2018), who revised the three specimens collected by J.K., confirmed his tentative identifications of the plants as *M. funckii*, having noted the dioicy in two of the specimens which bore gametangia.

With respect to the distinctive morphology of the plants and molecular differences from all other previously molecularly investigated species of *Marsupella*, we describe in this paper the species as new to science. The discovery on Serra de Monchique of a novel species, employing integrative taxonomic techniques [11], is not unprecedented; the mosses *Coscinodon monchiquensis* R.D. Porley, Ochyra & Ignatova and *Neodicranella hamulosa* R.D. Porley, Fedosov & Plášek were also recently described from the region [12,13], underlining the bryologically underexplored nature of the territory. By contrast, the more northern localities are considered bryologically much better worked, particularly in the case of Serra da Estrela [14] and Peneda-Gerês National Park [6,15,16,17]; Serra do Caramulo is, however, bryologically less well known [18].

## 2. Results

### 2.1. Molecular Data

Based on both ITS and *trnF–trnT* data, accessions of the Portuguese plants which are described below as a new species, *Marsupella lusitanica*, show little variation in ITS (two A/G substitutions in ITS1 in one of four analyzed accessions, two ITS1 substitutions further downstream in another accession, two one-base indels in two different poly-C, resp. poly-G regions of ITS2 in two of six accessions) and no variation in *trnF–trnL* region (*trnL–trnT* region was retrieved for only one accession). On the contrary, the sequences differ in eleven-point substitutions and three 2–8 bp indels from *M. profunda* only in ITS2, the only common region analyzed. Multiple substitutions and indels in both *trnF–trnT* and ITS regions differentiate the newly discovered species from other lineages of sect. *Ustulatae* Müll. Frib. ex R.M. Schust. as well.

**Figure 1:**
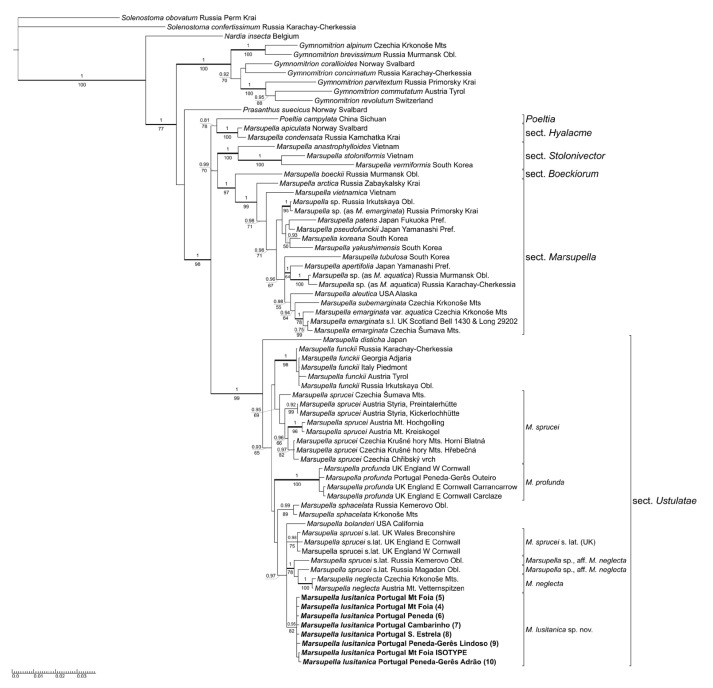
Consensus tree from the Bayesian inference of concatenated ITS and *trnF–trnT* data, partitioned between ITS and *trnF–trnT* nucleotide data and standard data from indels coded by a simple coding method [19]. Accessions of *Marsupella lusitanica* are printed in bold. Support values representing posterior probabilities from Bayesian analysis are plotted above branches, while bootstrap support values from Maximum Likelihood analysis of the equally partitioned dataset appear below branches. Support values are shown only on branches receiving either PP > 0.9 or BS > 70. Branches which received maximum support from at least one of the analyses and accessions of *Marsupella lusitanica* appear in bold.

Molecular synapomorphies of *M. lusitanica* are translated to the distinct pattern in tree topology summarizing the results of the phylogenetic analyses of both the concatenated data matrix (Figure 1) and the data from the analyses of individual markers, ITS and *trnL–trnT* (Supplementary Data, Figures S1 and S2, respectively). The well-supported lineage containing accessions of *M. lusitanica* is nested in a fully supported clade consisting of members of *Marsupella* sect. *Ustulatae* as understood by Bakalin et al. [3]: *M. disticha* Steph., *M. funckii*, *M. bolanderi* (Austin) Underw., *M. sphacelata* (Giesecke ex Lindenb.) Dumort., *M. profunda* and accessions of multiple lineages which are labeled as *M. sprucei* and *M. neglecta* (Limpr.) Lindb., a taxon generally treated as a variety or synonym of *M. sprucei* by recent authors [5,19,20,21]. This clade is unresolved in a polytomy with two other lineages, one corresponding to section *Hyalacme* (Lindb.) Bakalin & Fedosov (*M. apiculata* Schiffn., *M. condensata* (Ångstr. ex C. Hartm.) Lindb. ex Kaal.), and the other containing the clades corresponding to sections *Stolonicaulon* (N. Kitag.) Váňa, *Boeckiorum* Müll. Frib. ex R.M. Schust. and *Marsupella*. The latter section is the largest lineage containing the type of the genus, *M. emarginata*, and related species. A single accession of *Poeltia campylata* Grolle clusters with weak support with species of *Marsupella* sect. *Hyalacme*, while *Marsupella* including *Poeltia* appears very well supported, and *Prasanthus* Lindb., *Gymnomitrion* Corda and *Nardia* Gray branch off at successively deeper nodes. The affinities among lineages within sect. *Ustulatae* are poorly resolved except for the weakly supported sister relationship between *M. disticha* and the rest of the species, but accessions from most individual species are mostly well supported and show little variability. An interesting exception to this appears to be *M. sprucei*, accessions of which arise in multiple lineages, differently so in ITS and *trnF–T*-based datasets. ITS data (Figure S1) suggest differentiation into three lineages, the largest of them containing seven accessions from montane to alpine habitats of Central Europe and morphologically corresponding to *M. sprucei* in the strict sense, the second containing three accessions of plants labeled as *M. sprucei* from southwestern England and Wales, and the third containing accessions morphologically corresponding to *M. neglecta* from Central European alpine habitats (growing on soil in contrast to most occurrences of *M. sprucei* s.str. which were collected from epilithic habitats), and two molecularly somewhat divergent accessions from southern Siberia and the Russian Far East, for which morphological and ecological data are not known; the lineage including *M. neglecta* and two *M. sprucei* s.lat. accessions is nevertheless strongly supported. Chloroplast data (Figure S2) suggest a finer division into four lineages with unresolved mutual relationships (data for the UK specimens are unfortunately missing, but it is plausible to assume that they would form another distinct lineage); three of the lineages are formed by accessions of what appeared to form the nearly invariable ‘*M. sprucei* s.str.’ ITS lineage. Moreover, the two Russian accessions do not appear to be closely related based on their published *trnL–trnF* data, only the accession from Magadan region showing a moderately supported affinity with *M. neglecta*. The analysis of concatenated data (Figure 1) retains the ITS-based and morphology-supported clustering of *M. sprucei* s.str. (only weakly supported, PP 0.95/BS 69, given the chloroplast-based differences) and the clustering of *M. neglecta* with the Russian *M. sprucei* s.lat. accessions (with only a slightly weakened BS support) and resolves the British lineage of *M. sprucei* s.lat. distinct from both other *M. sprucei* s.lat. lineages.

### 2.2. Description of the New Species

**Figure 2:**
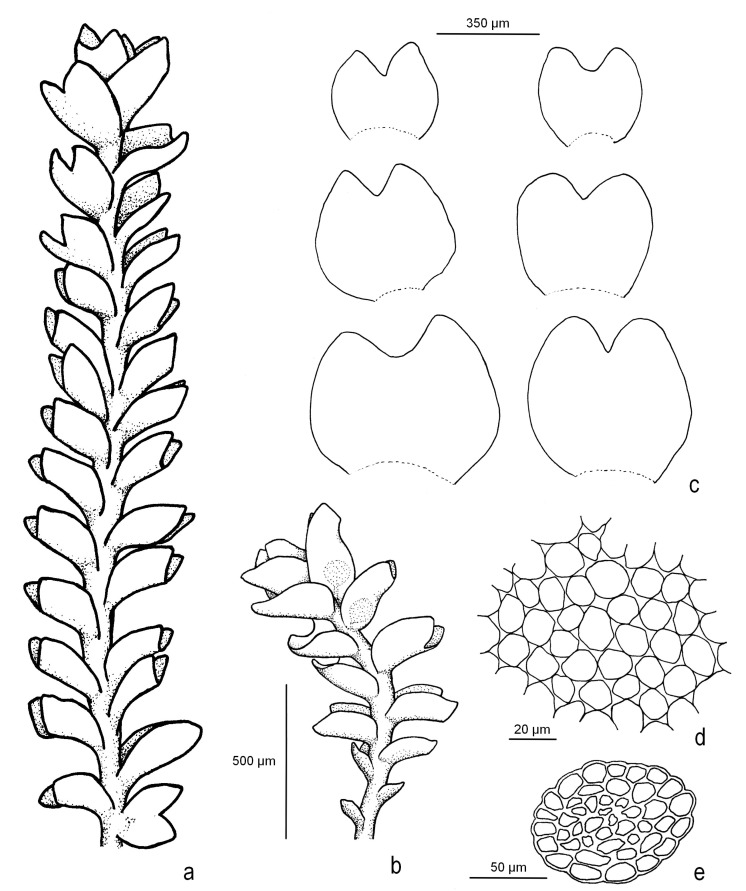
*Marsupella lusitanica* R.D. Porley & Jan Kučera (all from Porley *s.n.*, paratype 5): (**a**) sterile shoot, wet; (**b**) male plant, wet; (**c**) leaves; (**d**) cells in mid-lobe region; (**e**) cross-section of stem. Line drawing by Nick Hodgetts.

**Figure 3:**
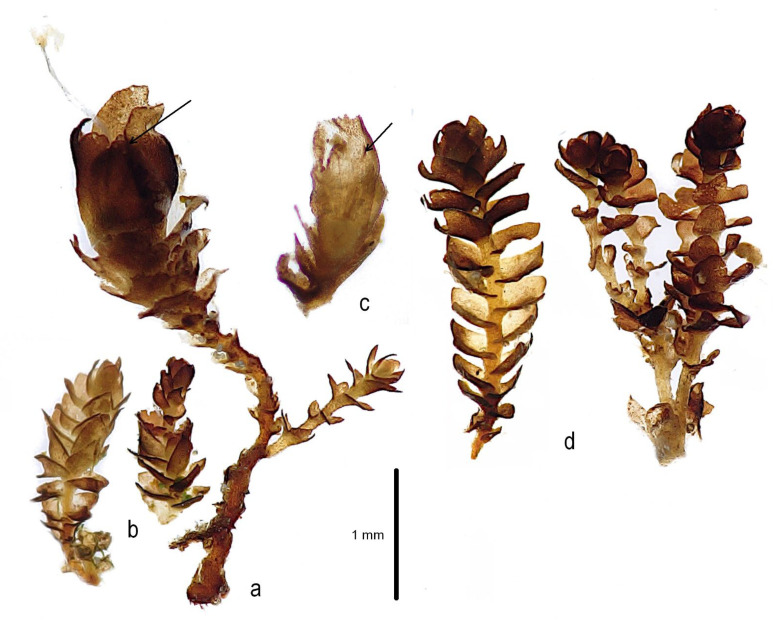
*Marsupella lusitanica* R.D. Porley & Jan Kučera, habit microphotographs: (**a**) female plant with perianth (arrow) and sporophyte; (**b**) two male shoots; (**c**) section of gynoecium showing the perigynium, enlarged bracts, and a smaller perianth (arrow) inside; (**d**) two sterile shoots. (**a**,**c**) from Kučera *10623*, paratype 7, (**b**) from Kučera *10685*, paratype 8, (**d**) from holotype, Porley *s.n.*

**Figure 4:**
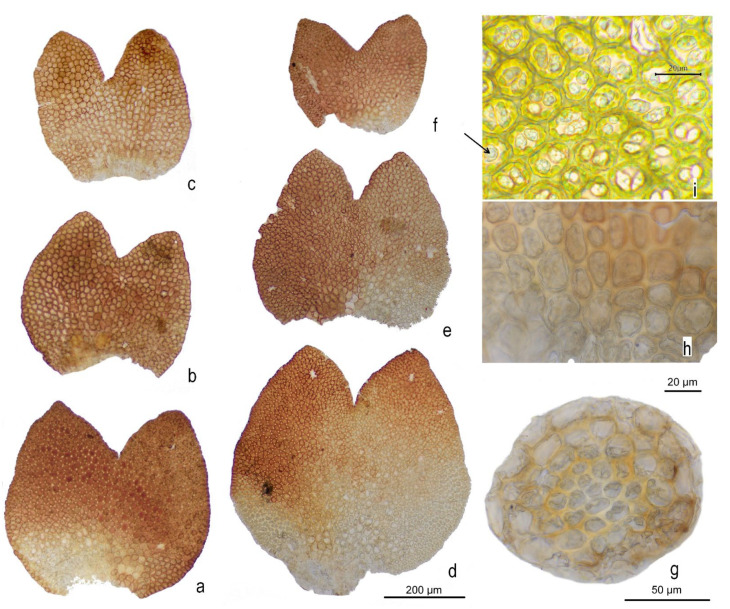
*Marsupella lusitanica* R.D. Porley & Jan Kučera, detail microphotographs: (**a**–**f**) vegetative leaves; (**g**) stem cross-section; (**h**) basal cells; (**i**) mid-lobe cells showing the oil bodies; arrow to biconcentric oil body. (**a**–**c**,**g**) from holotype, Porley *s.n.*, (**d**–**e**) from Kučera *10685*, paratype 8, (**f**,**h**) from Kučera *10623*, paratype 7, (**i**) from Porley *s.n.*, paratype 3.

***Marsupella lusitanica*** R.D. Porley & Jan Kučera, **sp. nov.** Figure 2, Figure 3 and Figure 4 and Figure S3.

**Etymology.** The specific epithet refers to the geographical provenance (from Latin *lusitanicus*, Lusitanian, Portuguese) from which the new species is currently only known.

**Diagnosis.** The species differs from other species of *Marsupella* sect. *Ustulatae* Müll. Frib. ex R.M. Schust. in the combination of dioicous distribution of gametangia, slightly wider than long, semiovate leaves mostly with a relatively open sinus (angle between lobes at base of sinus mostly 40–80°, angle at lobe tips mostly 90–120°), descending to 22–30% of leaf length, and subacute to obtuse leaf lobes with relatively small cells, 12–18 µm wide at the base of lobes and 8–13 µm wide at lobe margins.

**Type:** Portugal: Algarve, Faro District, Serra de Monchique, Mt. Fóia, 37.317894° N 8.592727° W, altitude 876 m, on inclined face of syenite rock on north-facing rocky outcrop on summit, with *Scapania compacta* (Roth) Dumort., *Grimmia trichophylla* Grev. and *Cephaloziella divaricata* (Spruce) Schiffn., 13 September 2015, with sporophytes, *leg*. R.D. Porley, LISU. Isotype CBFS (20843).

**Additional specimens examined (paratypes)**: (**1**) Algarve, Faro District, Fóia, Serra de Monchique, 37.317894° N 8.592727° W, altitude 876 m, syenite rock crevice on north-facing rocky outcrop on summit, 1 March 2019, with female gametangia, *leg*. R.D. Porley, herb. Porley; (**2**) *ibidem*, 13 March 2019, with female gametangia, *leg*. R.D. Porley, herb. Porley; (**3**) *ibidem*, in fissure of syenite block, N-facing, with crustose lichens, 19 January 2023, with archegonia, *leg*. R.D. Porley, herb. Porley; (**4**) *ibidem*, 37.319063° N 8.580492° W, 792 m, on syenite rock of terrace wall in crevice, N-facing aspect, with *Hypnum cupressiforme*, 3 December 2015, *leg*. R.D. Porley, herb. Porley, dupl. CBFS (20845); (**5**) *ibidem*, on syenite rock on terrace wall on sheltered north-facing slope, 21 November 2018, with antheridia, *leg*. R.D. Porley, herb. Porley, dupl. CBFS (20844); (**6**) Minho, Viana do Castelo District, Parque Nacional da Peneda-Gerês, Serra da Peneda: path Nossa Sra. da Peneda–Bouça dos Homens, at a brook 610 m WNW of the Na. Sra. da Peneda church, 41.977432° N 8.229002° W, ca. 920 m, shaded vertical face of a granitic boulder at the brook bank, N-facing, 4 July 2002, *leg*. J. Kučera *10,535* (CBFS); (**7**) Beira Alta, Viseu District, Serra do Caramulo, Cambarinho, on the edge of the laurel botanical reserve (ca. 0.6 km SE of the village), at a junction of tracks, 40.672565° N 8.201744° W, ca. 500 m, granitic stone by the track, light shade from the *Pinus pinaster–Eucalyptus* forest, 7 July 2002, *leg*. J. Kučera *10*,*623* (CBFS); (**8**) Beira Alta, Guarda District, Serra da Estrela, Loriga, path along the left riverbank of Ribeiro de Loriga, 130 m SW of the bridge 0.5 km SSW of the village center, 40.319168° N 7.697196° W, altitude 660 m, wall of granite stones, vertically, NW-or., not shaded, 9 July 2002, *leg*. J. Kučera *10*,*685* (CBFS); (**9**) Minho, Viana do Castelo District, Peneda-Gerês National Park, Lindoso Village, 41.867389° N 8.199222° W, castle wall, on granite rock, altitude 462 m, 11 June 2010, *leg*. D. Bell *251*, with D.G. Long (E); (**10**) Minho, Viana do Castelo, Peneda-Gerês National Park, Branda de Bordenca, SW of Adrão, 41.909333° N 8.264611° W, river valley, on granite wall by path, altitude 640 m, 11 June 2010, *leg*. D. Bell *247*, with D.G. Long (E).

**Description.***Plants* small, in low mats or patches closely attached to rock, brown to brownish-ochre, young growth yellowish-green, black to purplish-black when dry, lobes often more heavily pigmented than base of leaf giving a scorched appearance, somewhat glossy when dry, shoots erect (3–)4–5(–7) mm long and 0.4–1.0 mm wide, with intercalary branches and stolons with reduced leaves. *Rhizoids* few, scattered, smooth to slightly roughened, arising from stem only, colorless on younger stems and stolons, brownish or vinaceous. *Stem* cross-section ±0.14–0.22 mm wide, hyalodermis apparently lacking but outermost cortical cells enlarged, with less thickened walls, chlorophyllose only in young shoots, hyaline and eroded in older shoots, thick-walled medullary cells distinctly smaller. *Leaves* distichously arranged, channeled, transversely inserted, semiovate, ca. 0.30–0.52 mm long and 0.35–0.58 mm wide, widest below middle, 1.0–1.25× wider than long, imbricate distally, diminishing in size and distant proximally, margins plane, antical margin not or barely decurrent, patent to spreading, keel straight to slightly arched, lobes ± equal in size, apices subacute, occasionally obtuse, sometimes one lobe obtuse the other subacute on same leaf, sinus descending along mostly convex lobe margins to ca. 22–31(–40)% of leaf length, angle between lobes ca. 40–80° at base of sinus, 90–120(–140)° at lobe tips. *Leaf cells* isodiametric to somewhat oblong, (10–)12–18(–20) μm wide and (12–)14–21(–24) µm long, trigones large and bulging, marginal cells of lobes 10–18 μm long 8–13 μm wide, cuticle smooth. *Oil bodies* 2–3 per cell, spherical 4.5–7.5 μm to ovoid 10(–12) μm long, nearly filling cell lumen, containing 4–8 smaller oil droplets bound by an outer, only slightly irregular membrane; smaller part of the oil bodies biconcentric. *Dioicous*. *Androecia* in the upper part of stems mostly followed by vegetative leaves towards apex (intercalary), 0.7–0.75 mm wide, with 3–5 paired bracts with ventricose base, bracts ±530 μm wide with recurved antical margin, antheridial body ovoid–spherical, to 125 μm in diameter, antheridial stalks biseriate, to 95 μm long. *Gynoecia* terminal, to 1.0 mm wide, with subgynoecial innovations, bracts strongly concave, up to 850 μm wide, margins weakly recurved, perigynia well developed, perianths ovoid–conical, leptodermous, 0.2–0.5 mm high, over-topped by elongated upper bracts to 0.9 mm long, cells at mouth of perianth 35–40 μm long. *Capsule* spherical, *spores* almost smooth, (10–)11–12(–13) μm, elaters bispiral, 5–8 μm wide.

**Figure 5:**
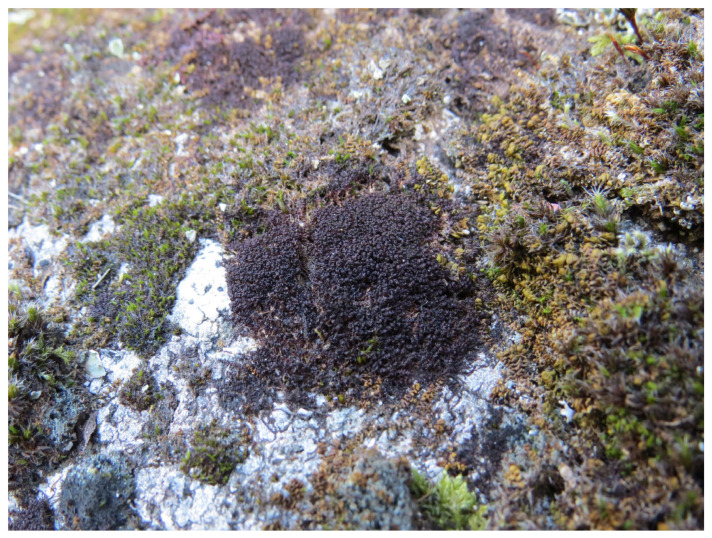
Dry habit of *Marsupella lusitanica* (paratype 1), showing the characteristic purplish black color, with *Scapania compacta*, *Racomitrium heterostichum* and *Grimmia trichophylla*. Photo by R.D. Porley, 1 March 2019.

**Differentiation.***Marsupella lusitanica* is characterized by a combination of relatively small size, dioicous sexuality, semi-ovate leaves with subacute to occasionally obtuse leaf lobes and moderately open sinus descending less than one-third of leaf length (Figure 4a–f), the glossy dark brown to purplish black color in the dry state (Figure 5; note, however the brownish appearance in the wet state, Figure S3), and the epilithic occurrence on acidic substrates in oceanic-montane but not high montane or alpine environments of westernmost Iberian Peninsula. With respect to the dioicy, which is a relatively rare condition in species of the sect. *Ustulatae*, shared by the wide-Holarctic-distributed *M. funckii* and *M. sphacelata* and the western North American *M. bolanderi*, *M. lusitanica* shares the general aspect of the smaller forms of the exceedingly variable (and possibly taxonomically heterogeneous) *M. funckii*. However, the typical form of *M. funckii* differs in leaves having acute widely triangular leaf lobes with wide (60–90°) and more deeply (1/3–1/2 of leaf length) descending sinus. The taxa described as *M. badensis* Schiffn. and *M. pygmaea* (Limpr.) Steph. are more similar to *M. lusitanica* in leaf shape, particularly the less deep sinus and somewhat ovate leaf lobes, but *M. badensis* is described as more robust (stems 1–2 cm tall as opposed to ca. 0.5(–0.7) cm tall stems of *M. lusitanica*), with the leaf sinus still more open and lobes with a mostly straight rather than convex line [22,23,24], and *M. pygmaea* being even smaller than *M. lusitanica*, with densely appressed leaves with acute lobes but larger leaf cells, 18–21(–25) µm [22,23,25]. The taxon described as *M. ramosa* Müll. Frib. shares the leaf shape of *M. badensis* but has substantially larger cells, 20–25 × 25–30 µm [22,23]. Obtuse lobes, the distribution in Portugal and apparently very similar ecology suggest the most likely confusion between *M. lusitanica* and *M. profunda*, but that species can safely be ruled out by its paroicous sexuality if gametangia are at hand, which fortunately is mostly the case in *M. profunda*. Gametophytic characters alone provide a less reliable differentiation; *M. profunda* usually has a different leaf shape, with leaves longer than wide (in *M. lusitanica* the leaves are typically 1.1–1.2× wide as long, Figure 4a–f), and broader, often lingulate lobes [26]. The sinus in *M. profunda* is often deeper and more distinctly U-shaped, and leaf cell dimension is marginally greater, although there is overlap. Perianth mouth cell length may be useful; for *M. lusitanica*, they were found to range between 25 and 40 μm, while measurements made in *M. profunda* indicate they are somewhat longer, ranging between 30 and 70 μm. However, only a small number (3–4) of perianths of *M. lusitanica* were available for study, whereas sporophytes with perianths are frequent in the paroicous *M. profunda.* Another dioicous species of the section, *M. sphacelata*, is usually substantially more robust, 1–5(–8) cm tall, with suborbicular to 2 mm long and wide leaves with obtuse lobes and very narrow sinus, and a distinct stem hyalodermis. The western North American *M. bolanderi* has a somewhat similar appearance, being a small reddish-brown dioicous species with suborbicular to semiovate leaves somewhat wider than long and a relatively deep (30–40%), wide sinus. Apart from the generally more open sinus, the median cells in *M. bolanderi* are larger (mostly 20–25 µm wide), and stem cortex cells thin-walled [27]. The small size, semi-ovate leaves and moderately wide sinus mostly between 20% and 30% of leaf length is seen in *M. sprucei* s.lat., which probably accommodates several taxa, as indicated by our molecular data. All taxa recognized within *M. sprucei* are nevertheless monoicous (mostly paroicous, although synoicous gametangia have been reported by Limpricht [25] and disputed in a follow-up account by Schiffner [28]). In *M. sprucei* s.str., fertile plants are distinctly clavate, with densely imbricate, julaceous leaves when dry, and the median leaf cells described as substantially larger than in *M. lusitanica*, about 20–25 µm, which is true also for our accessions from Czechia and a part of the Austrian collections (Köckinger *15,429*, *15,430*), which form two of the *trnF–T-*based lineages; plants from the other two Austrian collections which constitute another *trnF–T-*based lineage have variable cells, with most of them nevertheless rather significantly smaller, between 12 and 16 µm. In the taxon described as *M. neglecta*, which appears to be a distinct species based on our data, both the habit of plants (not particularly densely foliated, pectinate on sterile shoots, never clavate on fertile shoots) and leaf form and cell sizes are similar to *M. lusitanica*, but the lobes of vegetative leaves are sharply acute and the plants are clearly paroicous and nearly always fertile [25]. The differences among species of the sect. *Ustulatae* which are most likely to be confused with *M. lusitanica* at its localities are summarized in Table 1.

*Marsupella lusitanica* may on occasion also be confused with the dioicous *M. emarginata* growing in the same places; indeed, *M. emarginata* is frequently on the same rock outcrop (type locality) and terrace wall as *M. lusitanica*. However, normally it grows in more sheltered niches such as in hollows and under overhanging rocks, where it is usually more robust, but it also extends onto more exposed surfaces and onto protosoils surrounding outcropping rock. A study of these forms confirmed that when in the dry state, they never develop the intense shiny black coloration or the scorched appearance typical of *M. lusitanica* (Figure 5), and careful microscopic observation usually reveals at least traces of recurved antical leaf margins, which is generally the most useful discriminating character; note, however, that perigonial, perigynial and subperigynial bracts in *M. lusitanica* have distinctly recurved margins as well (Figure 3a,b). The leaf shape and sinus also differ. In *M. emarginata*, the leaves are orbicular or transversely elliptical (rarely very slightly longitudinally elliptical) in outline, with the widest place at about the middle of the leaf, with a markedly narrowed, at times cordate base, while the leaves of *M. lusitanica* are generally semi-ovate in outline, with the widest place below the middle (Figure 4a–f). The sinus is generally wider in *M. emarginata*, variously deep and the lobe shape is generally quite variable, but small forms of the species that are likely to be confused with *M. lusitanica* have the sinus mostly rectangular, and about one-third of the leaf length, with acute lobes.

## 3. Discussion

### 3.1. Molecular Affinities

Based on the molecular synapomorphies of the unidentified *Marsupella* plants from Serra de Monchique, Peneda-Gerês, Serra da Estrela and Serra do Caramulo, the plants should either be recognized as a distinct species within the sect. *Ustulatae*, or all species of the sect. *Ustulatae*, as understood by Bakalin et al. [3], should be synonymized as one species. Given the morphological differences with other currently recognized species of the section *Ustulatae* and the dioicy which otherwise only occurs in morphologically and molecularly different *M. bolanderi*, *M. funckii* and *M. sphacelata*, the description of the Portuguese plants as a new species is clearly the preferable option. At the same time, the discovery of the molecular heterogeneity of plants referrable to *M. sprucei* as understood in recent floras and checklists calls for a detailed study of these non-monophyletic units, the current understanding of which arose as a result of numerous synonymizations in earlier treatments as compared to the work of K. Müller [23]. The latter (the last comprehensive European liverwort flora) recognized at the species level *M. ustulata* Spruce and *M. sprucei*, differing from the former in substantially larger leaf cells; *M. neglecta* and *M. gracilis* (C. Massal. & Carestia) Pearson were recognized as identical at the varietal level under *M. ustulata*. The varietal status of *M. ustulata* and *M. neglecta* has been retained by Damsholt [29,30], and our molecular data, coupled with morphological and ecological features which match the original description [25], confirm that at least *M. neglecta* should clearly be recognized as a distinct species. The different signals from nuclear and plastid markers, along with sometimes overlapping morphological traits, do not allow for a clear separation of *M. sprucei* and *M. ustulata*; however, the number of studied accessions is so far too low for safely founded conclusions. A similar situation can be expected in taxa synonymized under *M. funckii*, where morphological variation may in the future be shown to be supported by molecular traits. The past synonymizations often occurred without much discussion. Thus, the synonymy of *M. ustulata* with *M. sprucei* was justified with the existence of intermediate cell sizes [20], the merger of *M. ramosa* with *M. funckii* was justified with the suggestion of the former being ‘a large-celled modification (probably polyploid)’ of the latter [31], and the synonymy of *M. pygmaea* and *M. badensis* received no justification at all [32,33]. On the other hand, the molecular support for the recognition of *M. aquatica*, as advocated by Vilnet et al. [7] and later authors, might be based on the neglect of cryptic diversity within the morphs corresponding to those taxa. European plants corresponding to *M. emarginata* and *M. aquatica* (both those selected by us and those deposited in GenBank by previous authors other than Vilnet et al. [7] and Bakalin et al. [3]) show either no differences at all or a different signal from ITS and plastid data (cf. Figure 1, Figures S1 and S2), and at the same time, they are molecularly clearly different from the plants (of mostly Russian provenance) sequenced under that name, despite us employing the most characteristic morphs corresponding to both taxa. This suggests the existence of two additional, morphologically probably nearly cryptic species in the *M. emarginata* complex, in addition to those revealed by Bakalin et al. [3], and simultaneously, the arguable specific distinctness of *M. aquatica*.

### 3.2. Habitat and Conservation

Serra de Monchique, situated at 37° latitude on the extreme southwest point of mainland Europe, Portugal, in Algarve province, lies within the Mediterranean macrobioclimate region of the Iberian Peninsula, defined by warm, wet winters with markedly dry summers. The highest point, Fóia, at 902 m, receives over 1000 mm of rainfall per year [34]; however, the E–W orientation of the massif and the close proximity to the North Atlantic Ocean results in warm, moist air currents bringing summer fog to N- and NW-facing aspects. The central part of the massif is known as the Monchique alkaline complex [35] and comprises rocks of alkaline feldspar and nepheline syenite, a silica under-saturated rock rich in alumina, alkalis, rare earths (e.g., Nd) and trace elements (e.g., Rb, Ba, Nb, Th and Pb), low in magnesium and iron [36,37], differentiating it from granite. Parque Nacional Peneda-Gerês is located in the north of Portugal in the provinces of Minho and Trás-os-Montes e Alto Douro. It is a mountainous area, dominated by granite rocks, near to the transition between the Mediterranean and Atlantic macrobioclimatic regions with a Euoceanic Continentality, and the diverse topography results in various microclimates that support vegetation characteristic of Mediterranean, Eurosiberian and Alpine phytogeographic environments. Of the summits, Peneda is at 1340 m and Gerês is at 1545 m, and receives precipitation levels in excess of 3000 mm per year at higher altitudes [15,16]. Serra da Estrela is located in central Portugal, in Beira Alta province, and is the highest point in mainland Portugal, at 1993 m, comprising a massive granite ridge. It experiences mainly a Mediterranean and Atlantic bioclimate, but also with Continental, Alpine and Boreal elements with a mean annual precipitation of about 2500 mm in its higher parts [14]. Serra do Caramulo, in Beira Alta, comprising granitic rocks, also experiences a Mediterranean and Atlantic bioclimate, with a mean annual precipitation of just over 2300 mm, but due to its lower altitude (the highest elevation Pico do Caramulinho at 1074 m), lacks alpine and continental elements [18].

**Figure 6:**
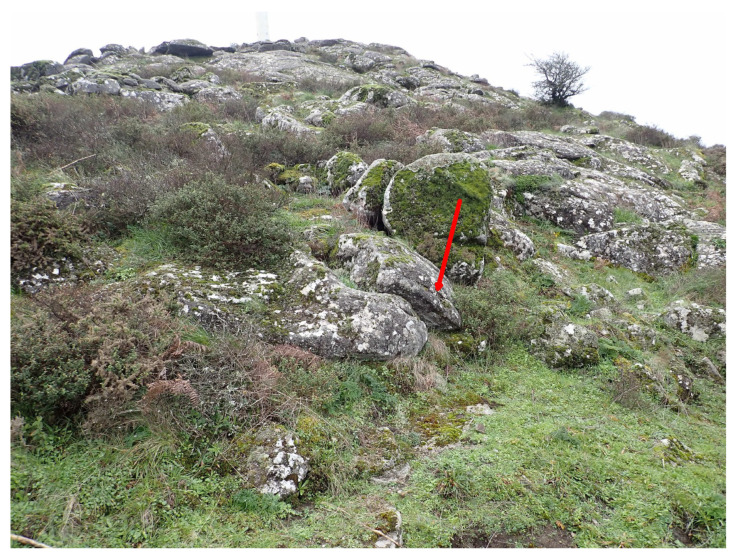
North-facing syenite rock outcrops on the summit of Fóia, Serra de Monchique, type locality of *Marsupella lusitanica*, one of the micropopulations (paratype 3) arrowed. Photo by R.D. Porley, 19 January 2023.

The habitat of the newly described species has been explored in detail only in Serra de Monchique. Here, *Marsupella lusitanica* occurs on a natural dome-shaped outcrop of syenite blocks and on an old terrace stone wall constructed from syenite rock on the north-facing flank of the mountain. It grows closely appressed to rock, typical of a pioneer species and characteristic of the genus [21]. At the type locality, only female plants have been detected. It occurs at several points, albeit in a small quantity, around the more humid base and lower levels of a north-facing ‘tor’ or outcrop at 876 m altitude (Figure 6), on exposed crustose and foliose lichen-covered rock surfaces and in shallow fissures (Figure 6 and Figure S4) typically mixed with *Grimmia trichophylla* Grev., *Hypnum cupressiforme* Hedw., *Scapania compacta* (Roth) Dumort., *Racomitrium heterostichum* (Hedw.) Brid., *Cephaloziella divaricata* (Sm.) Schiffn., *Frullania tamarisci* (L.) Dumort., *Cynodontium bruntonii* (Sm.) Bruch & Schimp. and, more rarely, *Frullania fragilifolia* (Taylor) Gottsche, Lindenb. & Nees. *Marsupella emarginata* is also present. The hyperoceanic microclimate of this outcrop is evidenced by the presence of the *Cisto crispi–Ulicetum minoris* gorse formation in which the lower woody stems of *Cistus salvifolius* L. support a rich epiphytic bryophyte community including *Antitrichia curtipendula* (Hedw.) Brid., *Ulota calvescens* Wilson and several *Orthotrichum* species [38]. However, significantly, also on the same outcrop is *Racomitrium lanuginosum* (Hedw.) Brid., a species in the circumpolar boreo-arctic montane element [39]. The other known locality (sites of paratypes 4, 5) on Serra de Monchique for *M. lusitanica* is ca. 1 km to the east on the north side of Fóia, at 784–792 m a.s.l., where it grows in at least two places on old neglected terraced north-facing moss-covered walls of syenite (Figure S5), partially overgrown by vegetation (*Crataegus monogyna* Jacq., *Pteridium aquilinum* (L.) Kuhn, *Rhododendron ponticum* subsp. *baeticum* (Boiss. & Reut.) Hand.-Mazz., *Rubus ulmifolius* Schott). This is the only site where male plants were detected; the majority of plants examined are female. *Marsupella emarginata* also occurs on the same wall. Much less detail is known about other sites in Portugal where the species was not collected intentionally, either supposed to be *M. sprucei* (DB’s collections from Peneda-Gerês) or *M. funckii* (J.K.’s collections from Peneda-Gerês, Serra da Estrela and Serra do Caramulo). In all three sites from the more northern Portuguese localities, the species was noted on vertical faces of granitic stones (either natural or from stone walls) in sheltered, moderately humid situations.

**Figure 7:**
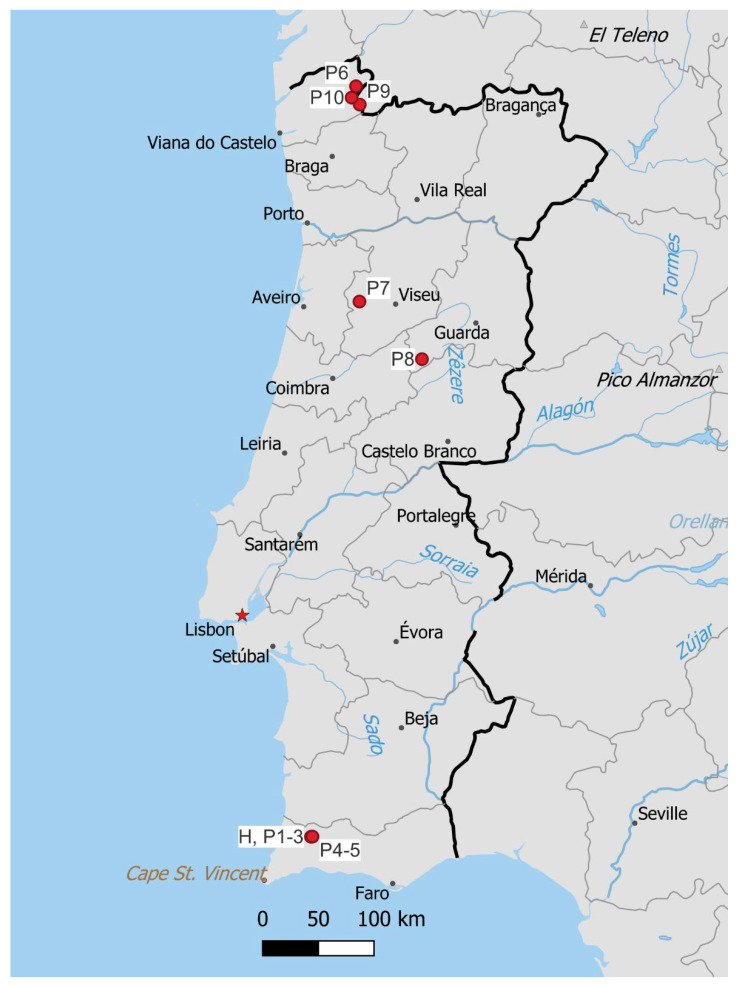
Known distribution of *Marsupella lusitanica*. H denotes the locality of holotype, P1–10 correspond to localities of paratypes 1–10. Made with Natural Earth.

Of the 16 European species of *Marsupella*, most show a northern distribution, in either the boreo-arctic montane or arctic-montane floristic element [39]. *Marsupella emarginata* shows a wider distribution, in the European Boreo-temperate element, and only one shows a much narrower range, *M. profunda*, classified as oceanic southern-temperate. In Portugal, *M. profunda* is reported from Parque Nacional Peneda-Gerês, Parque Natural do Alvão, Parque Natural da Serra de São Mamede and Parque Natural da Serra da Estrela; the latter site also supports *M. sprucei*, a taxon classified as Endangered [6] in Portugal. All these localities are in the high mountains of mid to northern Portugal. The currently known distribution of *M. lusitanica* (Figure 7) suggests a similar distribution pattern to *M. profunda* in Portugal, although it has not yet been recorded in Alvão and São Mamede natural parks, and, conversely, *M. profunda* has not been recorded in the Monchique mountains. These differences, however, rather suggest insufficient sampling effort in most Portuguese regions outside Peneda-Gerês and Serra da Estrela; it is most likely that further field sampling and revision of herbarium material will reveal additional populations. Given the broader distribution range of *M. profunda* that nevertheless has been revealed long after the initial discovery of the species, the possibility of endemicity of *M. lusitanica* to either Portugal or the Iberian Peninsula would be speculative and premature.

The population of *Marsupella lusitanica* in southern Portugal occurs within the 76,000 ha Monchique Natura 2000 Special Area of Conservation (SAC), yet it is debatable just how effective this designation is in protecting the biodiversity and natural communities and indeed the bryophytes, an issue highlighted in a recent horizon-scanning study to address fundamental questions in bryology [40]. The SAC has been extensively planted with *Eucalyptus* and pine and more ominously, wildfires are an ever-increasing threat compounded by rapid climate change and global warming [41]. A particularly devasting wildfire in 2018 covered over 27,000 ha on the northern and eastern flanks of the mountain, yet the impact on *M. lusitanica* and other bryophytes remains largely unknown. Rock outcrops are listed on Annex 1 of the European Community Habitats Directive (Siliceous rocky slopes with chasmophytic vegetation) yet are known to be particularly vulnerable to fire [42]. Parque Nacional Peneda-Gerês and Parque Natural da Serra da Estrela are both designated as SAC, and both areas have been impacted by extreme wildfire events, a situation that has been suggested as becoming the ‘new normal’ in Portugal [43]. *Marsupella profunda*, a species with an oceanic European and Macaronesian distribution, was assessed as ‘Vulnerable’ under criteria D1 in the last IUCN Red List of Threatened Species in Europe [44], meaning that while its population is not considered significantly declining, its size is small, estimated to be less than 1000 individual-equivalents. For *M. lusitanica*, the known extent of occurrence and area of occupation, as well as the documented population size, is apparently less, yet its actual distribution is obscured by greater uncertainty. The same inconclusiveness applies to possible trends in population dynamics. Even so, *M. lusitanica* arguably qualifies at a minimum for the ‘Vulnerable’ category worldwide (currently under both D1 and D2 criteria), prone to both effects of human activities and stochastic events within a short time period in an uncertain future. However, as the population is also possibly declining, it might potentially be assessed under B or C criteria, under which scenario it may be categorized as ‘Endangered’, even if the area of occupancy was between 10 and 500 km^2^ and the population size was estimated at between 250 and 2500 individual-equivalents, which may conceivably be a realistic approximation.

## 4. Materials and Methods

### 4.1. Molecular Investigation

Publicly accessible sequence data for most European *Marsupella* species and other genera of Gymnomitriaceae exist for the chloroplast region *trnL–trnF* and the nuclear ribosomal ITS. As for *M. profunda*, sequence data from ITS2, *trnH–psbA*, *rbcL* and *matK* were available from the unpublished DNA barcoding study on rare British bryophytes [10]. As there are limited publicly accessible *rbcL* or *trnH*-*psbA* data for *Marsupella*, no publicly accessible data for *matK*, and both *rbcL* and *trnH-psbA* are relatively uninformative for the genus, we focused our study on plastid *trnF–trnL* and nuclear ribosomal ITS to place the sequences of the new species into phylogenetic context. The dataset for the phylogenetic analysis was based on the available sequences in the GenBank database, which were retrieved using BLAST searches, and on the results of previously published molecular-phylogenetic studies in *Marsupella* by Bakalin et al. [3,4]. Sequencing of the *trnF–trnL* and ITS loci was carried out in the laboratories of the Faculty of Science, University of South Bohemia in České Budějovice, following the protocols for DNA isolation, amplification and sequencing described by Kučera et al. [45]. Where material allowed for the amplification of longer regions, we preferred to retrieve the chloroplast *trnF–trnL* including the adjacent variable *trnL–trnT* and *trnT–trnL* spacers. In these cases, amplification and sequencing employed the TabF [46] or our newly designed trnF-1F primer (5′– TGC CAG AAA CCA GAT TTG AAC TG –3′) as forward primers, and either the trnT-Bryo-R (5′– GGA GTC GAA CCG ATG ACC AT –3′) or rps4-22R (5′– GAG GTC CTC GAT AAC GNG ACA TAA –3′) as reverse primers (also designed by us). Newly generated sequences and others used for the context of the study are listed in the Appendix A.

Raw sequence reads were checked for reading errors and edited (primer complements and low-quality ends trimmed) and aligned with sequences downloaded from GenBank using the online interface of Mafft ver. 7 (https://mafft.cbrc.jp/alignment/server/, accessed on 18 March 2023 [47]) using the E-INS-i strategy with otherwise default options; the resulting alignment was checked and manually edited where necessary in Geneious Prime software (www.geneious.com, accessed on 18 March 2023). ITS and *trnF–trnT* matrices were evaluated independently, and as no topological conflict was discovered at supported nodes, a concatenated matrix was built and evaluated in phylogenetic context. The dataset was partitioned into ITS and *trnF–trnT* DNA data partitions and a standard data partition with indels scored using the simple-coding method [19]. Phylogenetic affinities were assessed using the Bayesian inference in MrBayes v. 3.2.7a [48] and Maximum Likelihood analysis using RAxML v. 8.2.12 [49], run at the cluster facilities of Metacentrum Virtual Organization (see Acknowledgements). The parameters for the analysis were set and trees from the analysis were summarized as described in Kučera et al. [45]. The trees were visualized using the TreeGraph2 software [50] and further edited graphically in Inkscape 1.2 (https://inkscape.org/, www.geneious.com) under the GPL license.

### 4.2. Morphological Investigation

The new species and specimens of species used for comparison were examined by standard morphological methods employing light microscopy and consultation of relevant morphological treatments and floras [9,22,23,29,51]. Emphasis was placed on characters most likely to provide distinction from other species in section *Ustulatae* and *Marsupella emarginata* from the same biogeographic region. The material collected and investigated by R.D. Porley is preserved in his personal herbarium (herb. Porley), the material investigated by J. Kučera is filed in herbarium CBFS and the material collected by D. Bell is kept in herbarium E. Abbreviations of herbaria follow the Index Herbariorum database (https://sweetgum.nybg.org/science/ih/, accessed on 18 March 2023).

## Figures and Tables

**Table 1:** Comparison of selected character traits among *Marsupella sprucei*, *M. funckii*, *M. profunda* and *M. lusitanica*.

Character	*M. sprucei s.lat.*	*M. funckii s.lat.*	*M. profunda*	*M. lusitanica*
Leaf shape	Distinctly longer than wide to slightly wider than long	Slightly to distinctly wider than long, seldom longer than wide	Mostly longer than wide	Mostly longer than wide
Sinus angle	50–100°	50–70°	(20–)40–60(–80)°	45–80° at base of sinus, 100–135°at lobe tips
Sinus depth	(1/5–)1/4–1/3(–1/2)	1/3–1/2 (*M. funckii* s.str.,), 1/4–1/5 (M. badensis)	(1/5–)1/3–2/5	1/5–1/3(–2/5) of leaf length
Lobe apex	Acute to narrowly rounded, or seldom ovate and more broadly rounded	Acute or subacute	Obtuse or subobtuse, occ. subacute, often broadly ovate or lingulate	Subacute to occasionally obtuse
Leaf cell size	12–18 μm (*M. neglecta*), 20–28 µm (*M. sprucei*)	12–18 μm (to 25 µm in *M. pygmaea*, to 30 µm in *M. ramosa*)	12–19 μm	(10)12–19(24) μm
Leafy shoot width	0.3–0.6(0.8) mm	0.3–0.6(0.8) mm (–1.1 mm in *M. badensis*)	0.4–1.0 mm	0.4–1.0 mm
Sexuality	Paroicous (rarely synoicous)	Dioicous	Paroicous	Dioicous
Color	Brownish green to dark brown, red or reddish black, seldom almost black	Brownish green to dark brown, reddish brown or almost black	Brownish green to dark brown, red or reddish black, seldom almost black and glossy when dry	Brown to brownish-ochre, new growth yellowish-green, to reddish-black and glossy when dry

## Data Availability

DNA sequences are available on the GenBank database and all authors agree with MDPI Research Data Policies.

## Supplementary Materials

The following supporting information can be downloaded at: https://www.mdpi.com/article/10.3390/plants12071468/s1, Figure S1: Consensus tree from the Bayesian inference of ITS data; Figure S2: Consensus tree from the Bayesian inference of plastid *trnF–trnT* data; Figure S3: Wet habit of *Marsupella lusitanica*; Figure S4: Detail of a site with a patch of *M. lusitanica*; Figure S5: Locality of paratypes 4 and 5.

## Appendix A

**Table A1:** Voucher information and GenBank accession numbers (ordered *trnL–trnF*(–*trnT*), ITS) of the specimens used in the molecular study. Newly obtained sequences are printed in bold. Accessions used in the study of Bell are identified using BOLD Process IDs which appear in the final BOLD and GenBank records upon their release.

**Species**	**Voucher Information**	**GenBank Accession Number**	
		**ITS**	***trnF–trnL* (*trnT*)**
*Gymnomitrion alpinum*	Czech Republic: Krkonoše Mts., Úpská jáma, *Kučera* 21057 (CBFS)	**OQ474588**	**OQ507748**
*Gymnomitrion brevissimum*	Russia: Murmansk Obl., *N. Konstantinova* G 8171 (KPABG)	EU791833	EU791711
*Gymnomitrion commutatum*	Austria: Tyrol, Mt Kreuzjoch, *Kučera* 18862 (CBFS)	**OQ474596**	**OQ507758**
*Gymnomitrion concinnatum*	Russia: Caucasus, Karachay-Cherkessia, *N. Konstantinova* K 465a-05 (KPABG)	EU791831	EU791710
*Gymnomitrion corallioides*	Norway: Svalbard, *N. Konstantinova* 155-04 (KPABG)	EU791826	EU791705
*Gymnomitrion parvitextum*	Russia: Primorsky Krai, *Mamontov* 170-1-10 (KPABG)	MF521472	MF521482
*Gymnomitrion revolutum*	Switzerland, *Kiebacher* 2235 (LE)	OQ024235	OQ029671
*Marsupella aleutica*	USA: Alaska, *Schofield* 103958 (MO)	MH826408	MH822632
*Marsupella anastrophylloides*	Vietnam: Hà Giang Prov., *Bakalin & Klimova*, V-15-6-20 (VBGI)	OM480746	OM489480
*Marsupella apertifolia*	Japan: Yamanashi Pref., *Bakalin* J-87-25-15 (VBGI)	MH539835	MH539892
*Marsupella apiculata*	Norway: Svalbard, *N. Konstantinova* K 93-1-06 (KPABG)	EU791819	EU791699
*Marsupella arctica*	Russia: Zabaykalsky Krai, Kodar Range, *Mamontov* 2-July-2013 (CBFS)	**OQ474591**	**OQ507752**
*Marsupella boeckii*	Russia: Murmansk Obl., Revda, *Kučera* 11525 (CBFS)	**OQ474590**	**OQ507751**
*Marsupella bolanderi*	USA: California, Santa Barbara, Santa Yen Mts., 38802 (KPABG)	MF521463	MF521475
*Marsupella condensata*	Russia: Kamchatka Krai, *Bakalin* K-60-30-15 (VGBI)	MH539844	MH539901
*Marsupella disticha*	Japan: *Deguchi & Yamaguch*i, Bryophytes of Asia 170 (KPABG)	EU791824	EU791703
*Marsupella emarginata*	Czech Rep.: Šumava Mts., Mt. Plechý, *Kučera* 23914 (CBFS)	OQ474584	OQ507743
*Marsupella emarginata*	UK: Scotland, *Bell* 1430	OL434465	–
*Marsupella emarginata* var. *aquatica*	UK: Scotland, *Long* 29202 (E)	–	KF942959
*Marsupella emarginata* var. *aquatica*	Czech Rep.: Krkonoše Mts., Úpská jáma, *Kučera* 20264 (CBFS)	**OQ474583**	**OQ507742**
*Marsupella funckii*	Russia: Caucasus, Karachay-Cherkessia, *N. Konstantinova* K 516-1-05 (KPABG)	EU791820	EU791700
*Marsupella funckii*	Georgia: Adjaria, *Bakalin* G-17-2-13 (VGBI)	MH539854	MH539911
*Marsupella funckii*	Italy: Piedmont, Ghiffa, *Kučera* 15227 (CBFS)	**OQ474581**	**OQ507740**
*Marsupella funckii*	Austria: Tyrol, Zillergrund, *Kučera* 18904 (CBFS)	**OQ474587**	**OQ507747**
*Marsupella funckii*	Russia: Irkutskaya Obl., Pik Taltsinskyi, *Kučera* 20642 (CBFS)	**OQ474593**	**OQ507754**
*Marsupella koreana*	South Korea: Gyeongsang Prov., *Bakalin* kor-23-18-15 (VGBI)	MH539850	MH539907
*Marsupella lusitanica*, **isotype**	Portugal: Algarve, Mt. Fóia, *Porley s.n.* 13-Sep-2018 (CBFS:20843)	**OQ474573**	**OQ507732**
*Marsupella lusitanica*	Portugal: Algarve, Mt. Fóia, *Porley s.n.* 21-Nov-2018 (CBFS:20844)	**OQ474574**	**OQ507733**
*Marsupella lusitanica*	Portugal: Algarve, Mt. Fóia, *Porley s.n.* 3-Dec-2015 (CBFS:20844)	–	**OQ507746**
*Marsupella lusitanica*	Portugal: Minho, Peneda-Gerês NP, Peneda, *Kučera* 10535 (CBFS)	**OQ474592**	**OQ507753**
*Marsupella lusitanica*	Portugal: Beira Alta, Campia, Cambarinho, *Kučera* 10623 (CBFS)	–	**OQ507756**
*Marsupella lusitanica*	Portugal: Beira Alta, Loriga, *Kučera* 10685 (CBFS)	**OQ474595**	**OQ507757**
*Marsupella lusitanica*	Portugal: Minho, Peneda-Gerês NP, Lindoso, *Bell* 257 (E)	**LWT1248-22**	–
*Marsupella lusitanica*	Portugal: Minho, Peneda-Gerês NP, Adrão, *Bell* 241 (E)	**LWT1269-22**	–
*Marsupella neglecta*	Czech Rep.: Krkonoše Mts., Mt. Sněžka, *Kučera* 18476 (CBFS)	**OQ474575**	**OQ507734**
*Marsupella neglecta*	Austria: Styria, Vetternspitzen, *Kučera* 14668 (CBFS)	**OQ474594**	**OQ507755**
*Marsupella patens*	Japan: Fukuoka Pref., *Bakalin* J-7-26a-14 (VBGI)	MH539846	MH539903
*Marsupella profunda*	Portugal: Minho, Peneda-Gerês, Outeiro, *Bell* 154 (E)	**LWT1242-22**	–
*Marsupella profunda*	UK: England, vc2, Carrancarrow, *Callaghan* 1069 (E)	**LWT1249-22**	–
*Marsupella profunda*	UK: England, vc2, Carclaze, *Holyoak* 09-47	**LWT1245-22**	–
*Marsupella profunda*	UK: England, vc1, Lower Bostraze, *Holyoak* 09-40	**LWT1246-22**	–
*Marsupella pseudofunckii*	Japan: Yamanashi Pref., *Bakalin* J-88-23-15 (VBGI)	MH539852	MH539909
*Marsupella* sp. (as *M. aquatica*)	Russia: Murmansk Obl., *Konstantinova* 152/5-87 (KPABG)	EU791813	AF519201
*Marsupella* sp. (as *M. aquatica*)	Russia: Caucasus, Karachay-Cherkessia, *N. Konstantinova* K 517-4-05 (KPABG)	EU791814	EU791694
*Marsupella* sp. (as *M. emarginata*)	Russia: Primorsky Krai, *Bakalin & Klimova*, Prim-16-14-16 (VBGI)	MH539848	MH539905
*Marsupella* sp.	Russia: Irkutskaya Obl., Snezhnaya River valley, *Kučera* 20641 (CBFS)	**OQ474582**	**OQ507741**
*Marsupella sphacelata*	Russia: Kemerovo Obl., *N. Konstantinova* 65/1-00 (KPABG)	EU791821	AF519200
*Marsupella sphacelata*	Czech Rep.: Krkonoše Mts., Úpská jáma, *Kučera* 21077 (CBFS)	**OQ474597**	**OQ507759**
*Marsupella sprucei*	Czech Rep.: Šumava Mts., Mt. Plechý, *F. Müller* s.n. 3-October-2007 (DR, dupl. CBFS:20918)	**OQ474576**	**OQ507735**
*Marsupella sprucei*	Czech Rep.: Krušné hory Mts., Horní Blatná, *F. Müller* s.n. 18-September-2018 (DR, dupl. CBFS:20919)	**OQ474577**	**OQ507736**
*Marsupella sprucei*	Czech Rep.: Krušné hory Mts., Hřebečná, *S. Biedermann* s.n. 18-September-2018 (DR, dupl. CBFS:20920)	**OQ474578**	**OQ507737**
*Marsupella sprucei*	Czech Rep.: Děčín distr., Chřibský vrch, *F. Müller* s.n. 17-July-2019 (DR, dupl. CBFS:20965)	**OQ474579**	**OQ507738**
*Marsupella sprucei*	Austria: Styria, Preintalerhütte, *Köckinger* 15430 (CBFS)	**OQ474585**	**OQ507744**
*Marsupella sprucei*	Austria: Styria, Kickerlochhütte, *Köckinger* 15429 (CBFS)	**OQ474586**	**OQ507745**
*Marsupella sprucei*	Austria: Salzburg, Mt. Hochgolling, *Kučera* 9363 (CBFS)	OQ474589	**OQ507749**
*Marsupella sprucei*	Austria: Styria, Mt. Kreiskogel, *Kučera* 6391 (CBFS)	–	**OQ507750**
*Marsupella sprucei* s.lat.	Russia: Kemerovo Obl., *N. Konstantinova* 54-1-00 (KPABG)	EU791823	HQ833031
*Marsupella sprucei* s.lat.	Russia: Magadan Obl., *Bakalin* mag-38-39-11 (VGBI)	MH539856	MH539913
*Marsupella sprucei* s.lat.	UK: England, vc1, Bakers’ Pit, *Bell* 531 (E)	**LWT1261-22**	–
*Marsupella sprucei* s.lat.	UK: England, vc2, St. Neot, *Holyoak* 99-465	**LWT1259-22**	–
*Marsupella sprucei* s.lat.	UK: Wales, vc42, Lower Neuadd Res., *Bell* 566 (E)	**LWT1262-22**	–
*Marsupella stoloniformis*	Vietnam: Lao Cai Prov., *Bakalin & Klimova*, V-11-11-17 (VBGI)	MH539859	MH539916
*Marsupella subemarginata*	Czech Rep.: Krkonoše Mts., Mt. Kotel, *Kučera* 23513 (CBFS)	**OQ474580**	**OQ507739**
*Marsupella vermiformis*	South Korea: Jeju Prov., *Choi* 120911 (VBGI)	MH539857	MH539914
*Marsupella vietnamica*	Vietnam: Lao Cai Prov., *Bakalin*, V-2-101-16 (VBGI)	MH539862	MH539919
*Marsupella tubulosa*	South Korea, South Gyeongsang Prov., *Bakalin* Kor-23-15-15 (VBGI)	MH539861	MH539918
*Marsupella yakushimensis*	South Korea, Gangwon Prov., *Choi* 8347 (VBGI)	MH539864	MH539921
*Nardia insecta*	Belgium, *N. Konstantinova* 102077 (KPABG)	EU791836	EU791714
*Poeltia campylata*	China: Sichuan, *Bakalin* 48-2-17, 37210 (VGBI)	MH580596	MH580593
*Prasanthus suecicus*	Norway: Svalbard, *N. Konstantinova* K 121-5-06 (KPABG)	EU791825	EU791704
*Solenostoma confertissimum*	Russia: Caucasus, Karachay-Cherkessia, *N. Konstantinova* K 459-8a-05 (KPABG)	GQ220774	GQ220758
*Solenostoma obovatum*	Russia: Perm Krai, *N. Konstantinova* K 324-1-04 (KPABG)	GQ220771	GQ220755
