# Supplementary material for: Marsupella lusitanica (Gymnomitriaceae, Marchantiophyta), a New Species of Sect. Ustulatae from Mountain Ranges of Portugal
Source: Plants (Basel). 2023 Mar 27;12(7):1468. doi: 10.3390/plants12071468 (PMC10097375; doi:10.3390/plants12071468)
Supplement: Supplementary file 1 [file plants-12-01468-s001.zip › plants-2282728-supplementary/Supplementary_Figure_Captions.pdf]

Supplementary Materials

Figure S1. Consensus tree from the Bayesian inference of ITS data, partitioned between nucleotide data and standard data from indels coded by simple-coding method [22]. Posterior probability support values >0.9 are plotted above branches, while bootstrap support values from Maximum Likelihood analysis of the equally partitioned dataset appear below branches. Branches which received maximum support from at least one of the analyses appear in bold.

Figure S2. Consensus tree from the Bayesian inference of plastid *trnF-trnT* data, partitioned between nucleotide and standard data from coded indels as in Fig. S1. The values above and below branches and their thickness correspond to BI and ML support values as in S1.

Figure S3. Wet habit of *Marsupella lusitanica*, from paratype 3. Photo R.D. Porley, 19 January 2023.

Figure S4. Detail of lichen and moss-covered rocks with a patch of *M. lusitanica* (paratype 3) arrowed. Photo R.D. Porley, 19 January 2023.

Figure S5. Terraced slopes on the north side of Mt Fóia, with stone walls which support the sites of *Marsupella lusitanica* paratypes 4 and 5. Photo R.D. Porley, 7 December 2018.
