# Supplementary figures and images for: Marsupella lusitanica (Gymnomitriaceae, Marchantiophyta), a New Species of Sect. Ustulatae from Mountain Ranges of Portugal
Source: Plants (Basel). 2023 Mar 27;12(7):1468. doi: 10.3390/plants12071468 (PMC10097375; doi:10.3390/plants12071468)

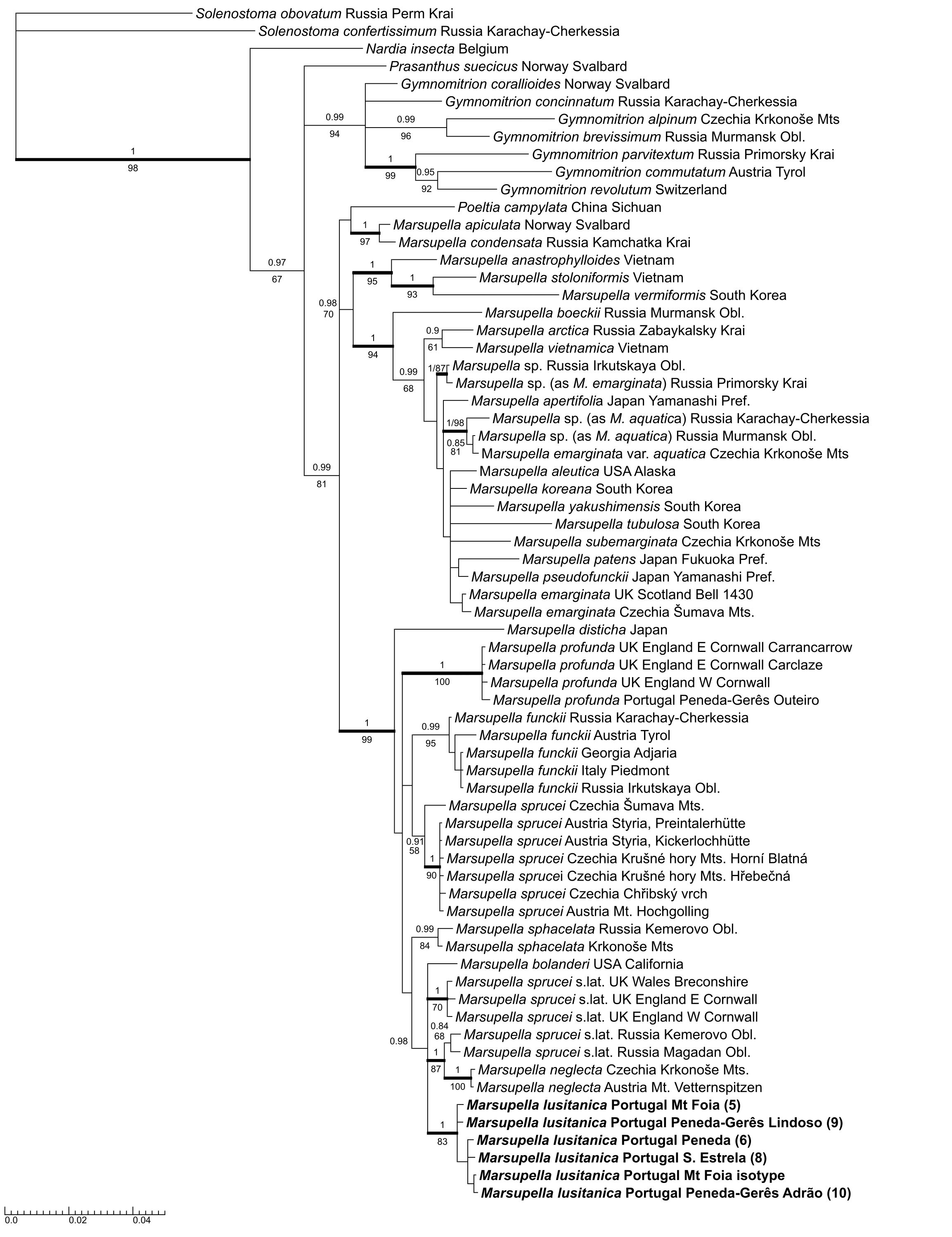

Supplement: Supplementary file 1 [file plants-12-01468-s001.zip › plants-2282728-supplementary/Suppl_Fig_S1_M_lusitanica_ITSsic.gif]

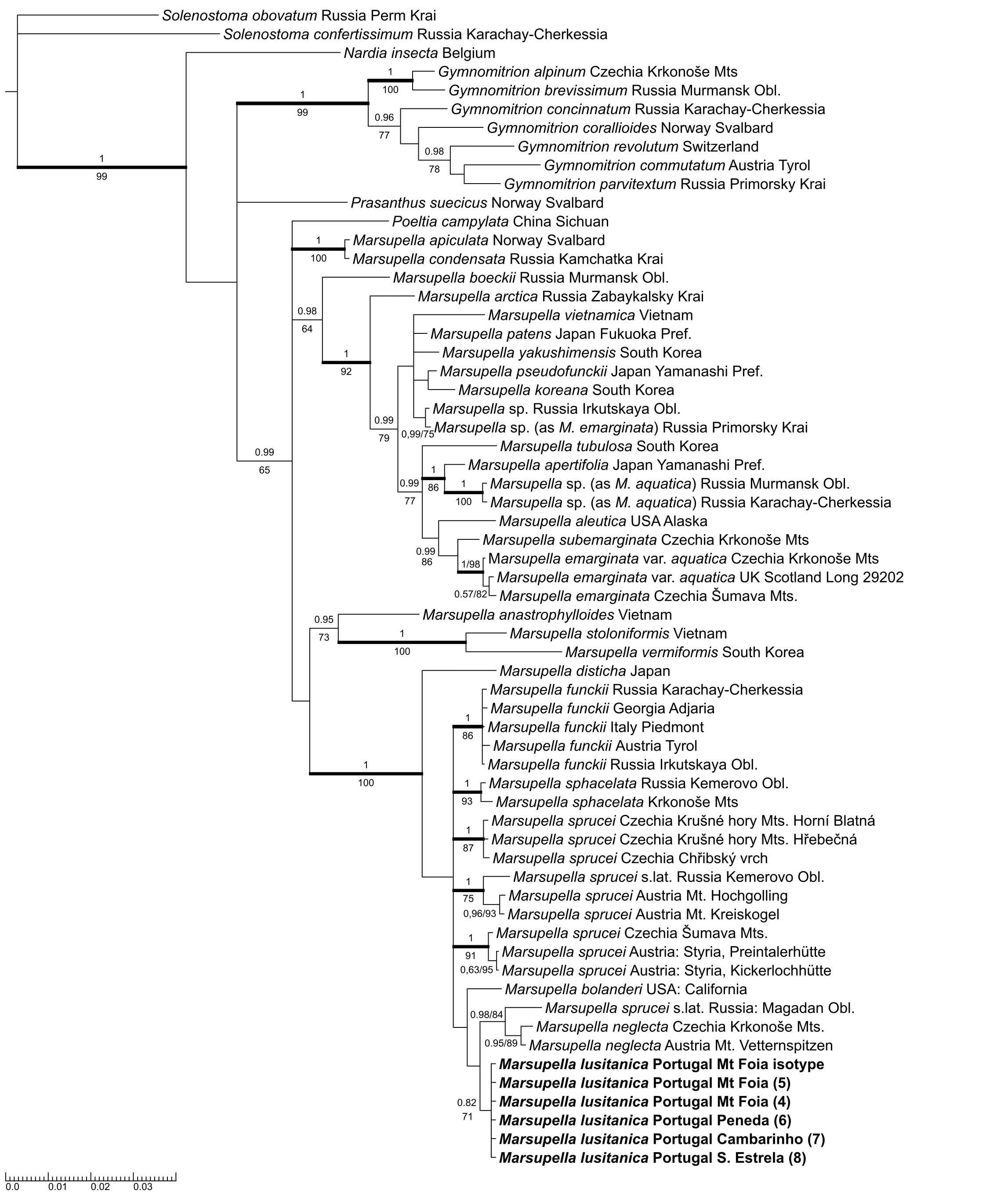

Supplement: Supplementary file 1 [file plants-12-01468-s001.zip › plants-2282728-supplementary/Suppl_Fig_S2_M_lusitanica_trnFT.gif]

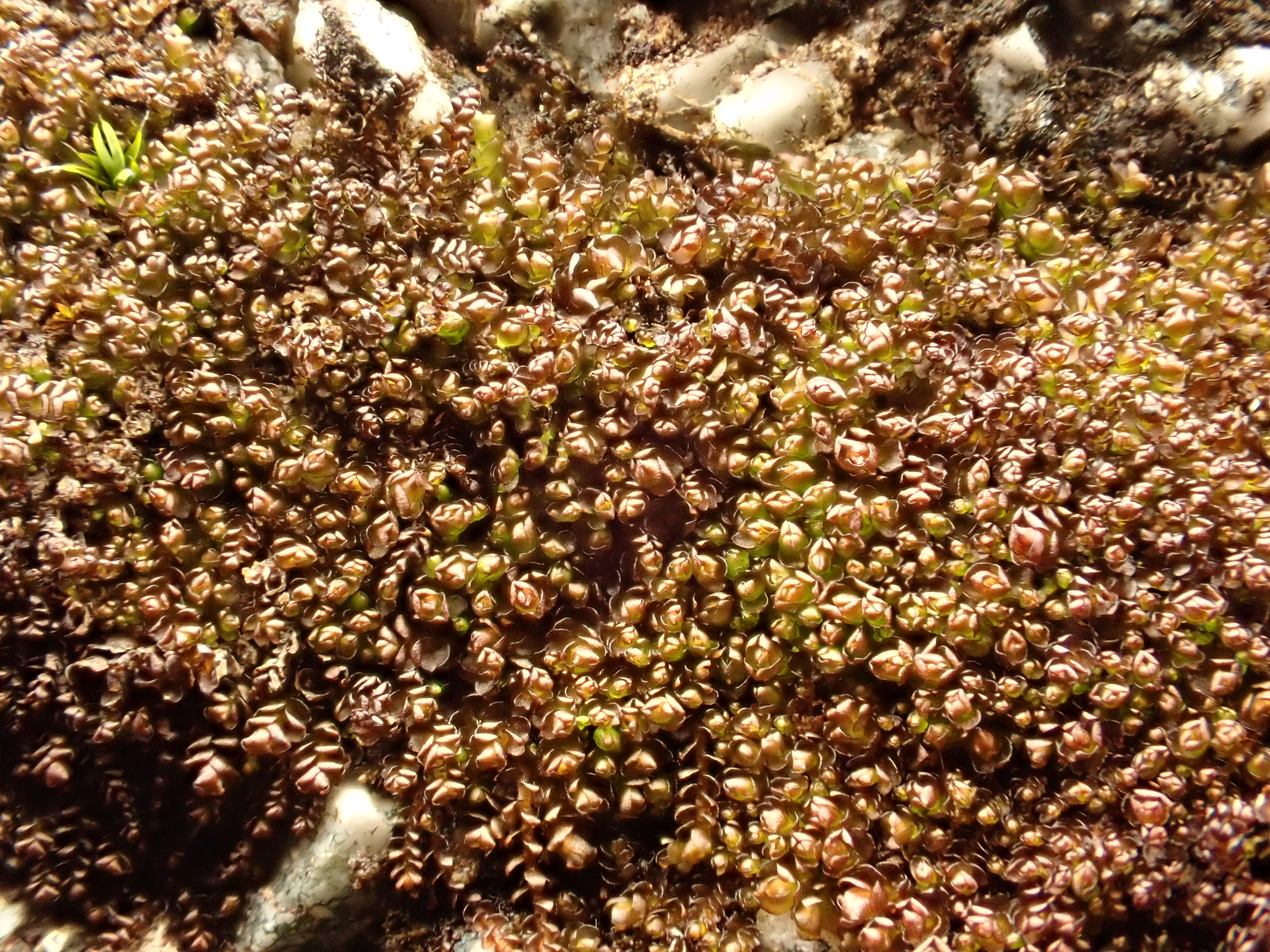

Supplement: Supplementary file 1 [file plants-12-01468-s001.zip › plants-2282728-supplementary/Suppl_Fig_S3_.Marsupella-lusitanica-habit_wet_19.1.23.jpg]

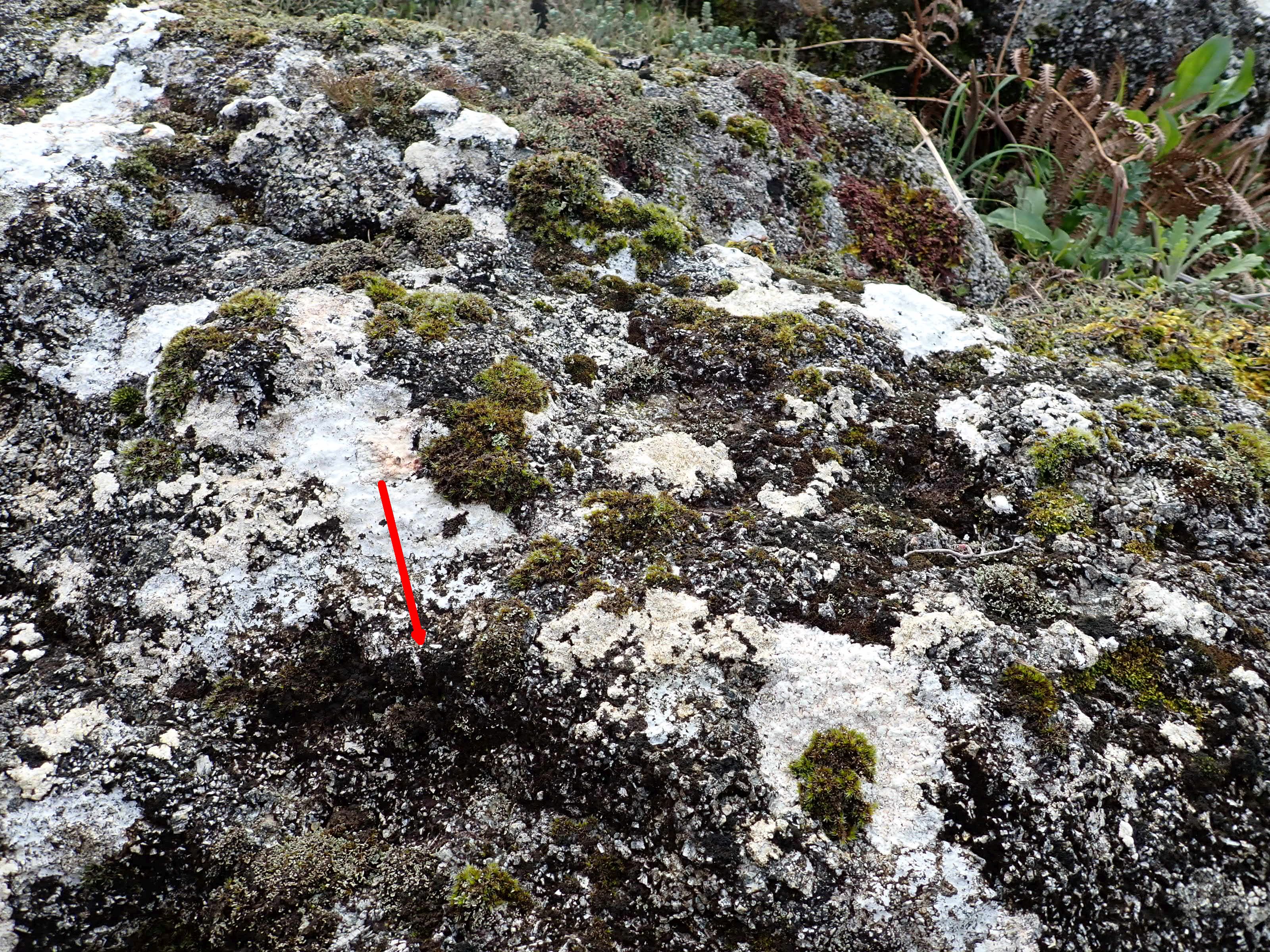

Supplement: Supplementary file 1 [file plants-12-01468-s001.zip › plants-2282728-supplementary/Suppl_Fig_S4_.Marsupella-lusitanica_habitat_19.1.23.jpg]

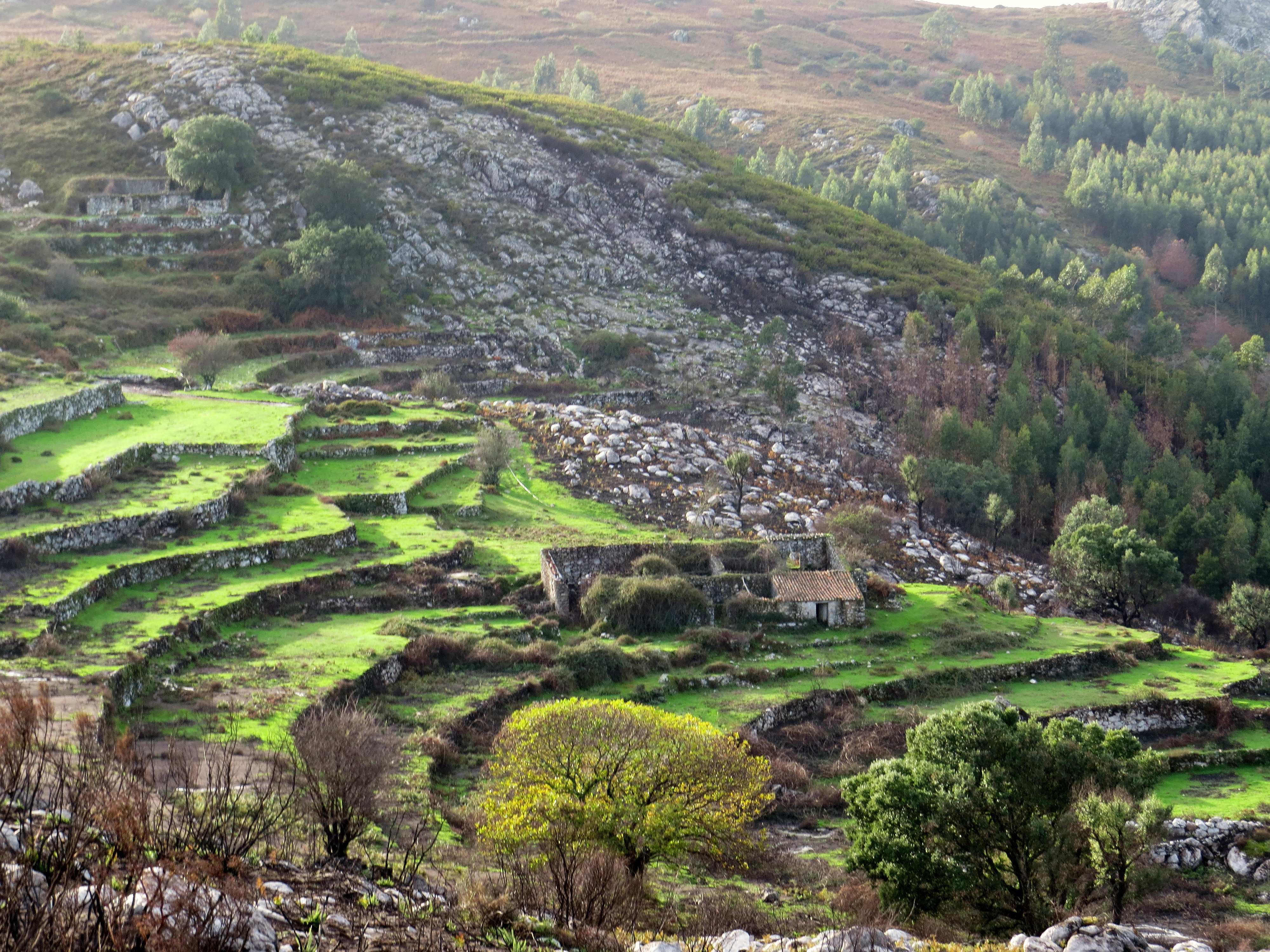

Supplement: Supplementary file 1 [file plants-12-01468-s001.zip › plants-2282728-supplementary/Suppl_Fig_S5_Foia-N-slopes-terraces_7Dec2018.jpg]
